# Association between osteoporosis and rheumatoid arthritis in women: a cross-sectional study

**DOI:** 10.1590/S1516-31802009000400007

**Published:** 2009-12-07

**Authors:** Karin Sedó Sarkis, Mariana Barbieri Salvador, Marcelo Medeiros Pinheiro, Raissa Gomes Silva, Cristiano Augusto Zerbini, Lígia Araújo Martini

**Affiliations:** 1 MSc. Nutritionist, Nutrition Department, Faculdade de Saúde Pública, Universidade de São Paulo, São Paulo, Brazil.; 2 Nutritionist, Nutrition Department, Faculdade de Saúde Pública, Universidade de São Paulo, São Paulo, Brazil.; 3 MD, PhD. Division of Rheumatology, Universidade Federal de São Paulo, São Paulo, Brazil.; 4 MD. Division of Rheumatology, Hospital Heliópolis, São Paulo, Brazil.; 5 MD, PhD. Division of Rheumatology, Hospital Heliópolis, São Paulo, Brazil.; 6 PhD. Nutritionist, Nutrition Department, Faculdade de Saúde Pública, Universidade de São Paulo, São Paulo, Brazil.

**Keywords:** Rheumatoid arthritis, Osteoporosis, Diet, Body composition, Physical activity, Parathyroid hormone, Artrite reumatóide, Osteoporose, Dieta, Composição corporal, Atividade física, Hormônio paratireóideo

## Abstract

**CONTEXT AND OBJECTIVES:**

Osteoporosis has frequently been observed in patients with rheumatoid arthritis. The present study was undertaken in order to evaluate factors associated with osteoporosis among women with rheumatoid arthritis.

**DESIGN AND SETTING:**

Cross-sectional study, carried out in a public hospital in São Paulo.

**METHODS:**

The participants were 83 women with rheumatoid arthritis (53.7 ± 10.0 years old). Bone mineral density (BMD) and body composition were measured by dual energy X-ray absorptiometry. The patients were divided into three groups according to BMD: group 1, normal BMD (n = 24); group 2, osteopenia (n = 38); and group 3, osteoporosis (n = 21). Tests were performed to compare differences in means and correlations, with adjustments for age, duration of disease and cumulative corticosteroid. The relationships between clinical factors, physical activity score, dietary intake, body composition and biochemical parameters were analyzed using linear regression models.

**RESULTS:**

Mean calcium, vitamin D and omega-6 intakes were lower than the recommendations. Associations were found between BMD and age, disease duration, parathyroid hormone concentration and fat intake. The linear regression model showed that being older, with more years of disease and lower weight were negatively correlated with BMD [Total femur = 0.552 + 0.06 (weight) + 0.019 (total physical activity) - 0.05 (age) - 0.003 (disease duration); R^2^ = 48.1; P < 0.001].

**CONCLUSION:**

The present study indicates that nutritional factors and body composition are associated with bone mass in women with rheumatoid arthritis.

## RESUMO

**CONTEXTO E OBJETIVO:**

A osteoporose é frequentemente observada em pacientes com artrite reumatoide (AR). O presente estudo foi realizado com o objetivo de avaliar fatores associados a osteoporose em mulheres com AR.

**TIPO DE ESTUDO E LOCAL:**

Estudo transversal realizado em um hospital público em São Paulo.

**MÉTODOS:**

Participaram 83 mulheres com AR (53.7 ± 10.0 anos). A densidade mineral óssea (DMO) e composição corporal foram mensuradas pelo método de densitômetro de dupla emissão com fonte de raios-X. Houve a divisão em grupos conforme a DMO: grupo 1 DMO normal (n = 24); grupo 2 osteopenia (n = 38) e grupo 3 osteoporose (n = 21). Foram realizados testes para comparar diferenças de médias e correlações ajustadas para idade, duração da doença e corticosteroide acumulado. A relação entre fatores clínicos, escore de atividade física, consumo alimentar, composição corporal e parâmetros bioquímicos foram analisados pelo modelo de regressão linear.

**RESULTADOS:**

A média de consumo de cálcio, vitamina D e ômega-6 esteve abaixo do recomendado. Houve associação entre DMO e idade, duração da doença, hormônio da paratireóide (PTH) e consumo de gordura. O modelo de regressão linear evidenciou que ser mais velho, apresentar mais anos de doença e menor peso correlacionam-se negativamente com a DMO. [Fêmur total = 0.552 + 0.06 (peso) + 0.019 (atividade física total) - 0.05 (idade) - 0.003 (duração da doença), R^2^ = 48.1; P < 0.001].

**CONCLUSÃO:**

O presente estudo indica que fatores nutricionais e de composição corporal estão associados com a massa óssea em mulheres com AR.

## INTRODUCTION

Rheumatoid arthritis is a chronic inflammatory and destructive joint disease that affects 0.5-1% of the world’s population and commonly leads to significant disability and consequent impairment of quality of life.^1^^,^^2^ It is two or three times more frequent in women than in men and can start at any age, with its peak incidence between the fourth and sixth decades of life.^3^

Generalized osteoporosis is an extra-articular complication of rheumatoid arthritis that results in increased risk of fractures and associated morbidity, mortality, and healthcare costs.^4^ The incidence of osteoporosis among patients with rheumatoid arthritis is 15-20% at the hip and spine.^5^

Bone metabolism in rheumatoid arthritis cases is altered by the chronic inflammatory process, via activation/inhibition of bone cell function,^6^ modification of body composition, corticosteroid use,^7^ diet^8^ and low levels of physical activity.

Moreover, during the active phase of the disease, elevated plasma concentrations of inflammatory cytokines, i.e. interleukin-1 (IL-1), interleukin-6 (IL-6) and tumor necrosis factor-alpha (TNF-a) lead to reductions in fat-free mass with a loss of body cell mass and consequent reduction in muscle strength.^9^ This loss may negatively affect bone mineral density (BMD), because lean mass has been reported to be a predictor of bone mass through its mechanical pull on the skeleton.^10^

Corticosteroid treatment presents a relative contribution, since it also induces osteoporosis by several mechanisms, such as through decreased calcium absorption, increased renal calcium excretion and inhibition of estrogen production in women, with direct and indirect effects on osteoblast and osteoclast function.^7^

The role of diet in rheumatoid arthritis and bone metabolism has been widely investigated, especially calcium, vitamin D, protein and lipid intake. The positive effects of adequate calcium and vitamin D intake on bone metabolism, through downregulation of serum parathyroid hormone (PTH) levels have been well established.^11^ It has also been well recognized that proteins have a beneficial effect on bone mass, inasmuch as bone tissue is formed by protein.^12^ A substantial fraction of the amino acids in bone collagen cannot be reutilized in new protein synthesis. Hence, bone turnover requires continuous intake of new protein.^13^

Some research has suggested that dietary lipids may also be important for bone health in rheumatoid arthritis cases, since there is a notable link between increased lipid peroxidation and bone loss.^14^^,^^15^ Also, two types of polyunsaturated fatty acids (w-3 and w-6) influence bone metabolism.^16^ Higher w-3 fatty acid intake enhances calcium absorption, decreases calcium loss and increases bone calcium.^17^^,^^18^^,^^19^ In addition, inhibition of cytokine production has been implicated as a potential mechanism for the favorable effects of fatty acids on bone.^20^^,^^21^ On the other hand, a higher w-6/w-3 ratio is associated with lower BMD^22^ while, in rheumatoid arthritis patients, a lower w-6/w-3 ratio suppresses the inflammatory cytokines,^23^^,^^24^ which could therefore benefit bone metabolism.

## OBJECTIVE

The present study was undertaken in order to evaluate factors associated with osteoporosis among women with rheumatoid arthritis.

## MATERIALS AND METHODS

### Subjects

This was a cross-sectional study with a convenience sample that was performed in a rheumatology outpatient clinic at a public hospital, Heliópolis Hospital, São Paulo, from June 2005 to January 2006. Patients under 18 years of age, male patients, pregnant women, patients who had diseases causing significant nutritional status impairment (neoplasia, end-stage renal failure, cardiovascular and gastrointestinal disease, such as aneurysm, angina, congestive heart failure, ischemic heart disease, stroke, inflammatory bowel disease, lactose intolerance and celiac disease) and patients with a history of chronic alcoholism were excluded.

One hundred and twenty women were evaluated. Thirty were excluded because they did not fulfill the necessary criteria for this study and another seven were excluded because they presented locomotion difficulty. Thus, 83 women who fulfilled the American College of Rheumatology^25^ criteria for rheumatoid arthritis and agreed to participate were included in the present analysis. Participants’ eligibility was assessed by a physician. The mean age (± standard deviation, SD) of the patients was 53.7 ± 10.0 years and they had had the disease for a mean of 7.7 ± 6.5 years. 

The subjects were divided into three groups in accordance with World Health Organization (WHO) criteria for low bone mass: group 1, normal BMD (n = 24); group 2, osteopenia (n = 38); and group 3, osteoporosis (n = 21).^26^ The estimated sample size was 44, based on a 1% prevalence of rheumatoid arthritis in the general population, standard error of 1.5% and type 1 (a) error of 0.05.^27^ The study protocol was approved by the ethics committees of Universidade de São Paulo and Heliópolis Hospital, and an informed consent statement was signed by each participant.

### Clinical data collection

Standardized questionnaires were used to ascertain demographic characteristics, medical history and use of oral glucocorticosteroids. Functional status was assessed using the Health Assessment Questionnaire (HAQ) score^28^ and disease activity was assessed using the Disease Activity Score-28 (DAS-28).^29^

### Dietary assessment

Dietary intake was assessed by using a validated food frequency questionnaire with 62 food items.^30^ This food questionnaire was administered by a trained nutritionist and provided information about food consumption over the preceding six months. The Dietary Analysis System (Dietsys) software, version 4.0 (National Cancer Institute, Bethesda, United States), was used to calculate nutrient intake. Mean daily intake, including total energy (kcal/day), total macronutrients, calcium, vitamin D and fatty acids were evaluated. The nutrients were adjusted for energy in accordance with Willett and Stampfer.^31^ Nutritional intakes were compared with the recommendations of the Food and Nutrition Board’s dietary reference intakes.^32^^,^^33^

### Physical activity assessment

Physical activity was assessed using the Baecke Questionnaire of Habitual Physical Activity.^34^ All subjects completed the questionnaires. Normal physical activity was assessed using three components: work, sport and leisure. All responses, with the exception of occupation activity and type of sport played, were precoded on a five-point scale, with descriptors ranging from never (1) to very often (5). Occupational activity was scored as low-level (1), middle-level (3) or high-level (5) activity. The sport score was calculated as (intensity code x duration code x code for proportion of the year) x 1.25. Each activity component could receive a maximum of five points, thereby giving a maximum of 15 for the physical activity index. Each index was rounded to the nearest tenth of a point.

### Anthropometry

The subjects were weighed on balance beam scales to the nearest 0.1 kg. Standing height was measured on the stadiometer of the balance, to the nearest centimeter. Body mass index was calculated as weight/height^2^ (kg/m^2^).

### Body composition and bone mineral density assessment

BMD measurements were performed on the lumbar spine (L1-L4) and femur by using dual energy X-ray absorptiometry (GE-Lunar Radiation Corporation, DPX IQ, Madison, United States). The device was calibrated daily and had a coefficient of variation of 1.5% for the spine and femur. Osteoporosis was defined as a T-Score £ -2.5, and osteopenia was defined as a T-Score between -1 and -2.5.^26^

Percentage of total body fat (%BF), total fat-free mass, and appendicular lean mass were measured using the same methodology (GE-Lunar Radiation Corporation, DPX IQ, Madison, United States). The relative skeletal muscle index (RSMI) was derived from the appendicular lean mass (kg) divided by the square of the height (m^2^). Sarcopenia was defined as RSMI of two standard deviations below the sex-specific mean for a young healthy reference population (< 5.45 kg/m^2^ for women).^35^

### Biochemical analysis

Blood samples were drawn from the patients after four hours of fasting. Serum intact parathyroid hormone (iPTH; normal range 15 - 65 pg/ml) was measured by using an electrochemiluminescence method. Serum total calcium (normal range 8.4-10.2 mg/dl) was measured by using a colorimetric assay and serum phosphorus (normal range 2.4-4.6 mg/dl) was measured by using UV mobility. C-reactive protein (CRP) was measured by means of latex agglutination (normal range: greater than 0.5 mg/dl), and the erythrocyte sedimentation rate was measured using the Westergren method (normal range: up to 10 mm in the first hour). Serum osteoprotegerin was determined using the Enzyme-Linked ImmunoSorbent Assay (ELISA) method (Alpco Diagnostics, Windham, United States) Seropositivity for rheumatoid factor was ascertained by means of the Singer-Plotz latex agglutination test. The analyses were performed at the hospital laboratory.

### Statistical analysis

Results were expressed as means and standard deviations. Statistical analyses were performed using SAS for Windows (Statistical Analysis System), version 8.02. (SAS Institute Inc, 1999-2001, Cary, United States) and the Statistical Package for the Social Sciences, version 11.5 for Windows (SPSS Inc, Chicago, United States). The sample power was calculated according to the single proportion test.^27^ The significance level taken was 5%.

Differences between groups were evaluated by means of analysis of variance (ANOVA). Analysis of covariance (ANCOVA) with age, duration of disease and cumulative prednisone use as covariates was also performed. Parametric and nonparametric tests were used in accordance with the type of variable distribution, for mean difference comparisons and correlation tests. Linear regression models were used to identify possible factors relating to BMD (dependent variable). The general and clinical features, dietary intake data, physical activity scores, body composition measurements and biochemical parameter concentrations were independent variables. All variables showing significant correlations with BMD were tested using a stepwise method. P-values of < 0.05 were considered significant.

## RESULTS

The demographic and clinical characteristics (mean ± standard deviation) of the 83 patients are reported in Table 1. The osteoporotic women were significantly older and had had the disease for longer times. Inflammatory activity was classified as moderate in all groups. No statistically significant differences were found in mean HAQ and total physical activity scores. A significant difference was observed regarding cumulative prednisone dose: women with osteopenia had higher cumulative doses than did those with normal BMD (P < 0.05). No significant differences were observed in current prednisone doses.

Body mass index (BMI), total fat-free mass, appendicular lean mass and body fat were lower in the osteoporosis BMD group (P < 0.05). Only one subject in the osteoporosis BMD group was classified as sarcopenic.

The dietary intakes are presented in Table 2. No statistically significant differences were found in mean daily energy, protein, calcium, vitamin D and fatty acid intake. On the other hand, mean daily fat intake was significantly higher among osteoporotic women than among others. The mean daily w-6/w-3 ratio was higher in the osteopenia group than in the normal BMD group (P < 0.05), and the mean daily carbohydrate intake was lower in the osteopenia BMD group than in the normal BMD group (P < 0.05). In all groups, mean macronutrient and w-3 intake were within the ranges proposed by the Food and Nutrition Board for dietary reference intakes.^32^ Mean calcium, vitamin D and w-6 intakes were lower than the proposed dietary reference intake values.^32^^,^^33^

Among the biochemical parameters (Table 3), the mean serum total calcium was higher in the normal BMD group than in the osteopenia group, despite remaining within the normal range. The serum phosphorus and iPTH were within their respective normal ranges, and there were no significant differences between the groups. The mean osteoprotegerin concentration was significantly higher among the women with osteoporosis.

The significant correlations between BMD and different variables are presented in Table 4. BMD presented negative correlations with age and disease duration at all sites evaluated. There was also a negative correlation between serum iPTH and BMD (P < 0.05, r = -0.24). Nevertheless, physical activity work index, total physical activity, BMI, lean mass and fat mass were positively correlated with BMD.

Table 5 presents the multiple linear regression models for lumbar spine, femoral neck and total femur BMD. Adjustments for possible interactions and potential confounders were made for vitamin D, calcium intake, weight, physical activity and corticosteroid use. It was found that age and disease duration were independent deleterious risk factors for BMD, at all skeletal sites. Fat intake and iPTH were also considered to be negative risk factors for lower spine BMD and femoral neck BMD, respectively. Weight had a positive effect on spine BMD. Weight, appendicular lean mass and total physical activity had a positive effect on femur BMD.

**Table 1.** General and clinical features of the population studied, according to bone mineral density (BMD)

Variable	Normal BMD	Osteopenia BMD	Osteoporosis BMD	P
	n = 24	n = 38	n = 21	
	Mean (SD)	Mean (SD)	Mean (SD)	
Age (years)	49.9 ± 9.6	52.1 ± 9.4	61.0 ± 7.8 ^*†^	< 0.001
Disease duration (years)	5.1 ± 4.5	8.0 ± 7.6	10.2 ± 5.5 ^*†^	0.030
BMI (kg/m^2^)	29.6 ± 5.7	26.8 ± 4.9	23.4 ± 3.6^*†^	< 0.001
DAS-28 (score)	4.8 ± 1.3	4.9 ± 1.2	4.5 ± 1.3	0.498
HAQ (score)	0.9 ± 0.7	1.1 ± 0.8	0.8 ± 0.7	0.368
Work index (score)	3.1 ±0.5	2.9 ± 0.5	2.6 ± 0.5	0.125
Sport index (score)	2.2 ± 0.6	2.0 ± 0.6	2.0 ± 0.5	0.273
Leisure index (score)	7.4 ± 1.5	6.7 ± 1.4	7.0 ± 1.8	0.326
Total physical activity index (score)	12.8 ± 1.9	11.7 ± 1.8	11.7 ± 2.2	0.171
Current prednisone (mg)	5.7 ± 4.7	5.7 ± 3.0	4.8 ± 3.4	0.586
Cumulative prednisone (mg)	2706.3 ± 2121.3	4219.0 ± 2272.8^‡^	3631.8 ± 2102.6	0.034
Lumbar spine BMD (g/cm^2^)	1.202 ± 0.098	1.019 ± 0.102^‡^	0.852 ± 0.096^*†^	< 0.001
Femoral neck BMD (g/cm^2^)	1.037 ± 0.119	0.847 ± 0.082^‡^	0.657 ± 0.085^*†^	< 0.001
Total femur BMD (g/cm^2^)	1.066 ± 0.110	0.879 ± 0.079^‡^	0.678 ± 0.078^*†^	< 0.001
Total fat-free mass (g)	44471.7 ± 4839.5	41407.7 ± 5157.2^‡^	37194.3 ± 4162.7^*†^	< 0.001
Appendicular lean mass (kg)	20934.6 ± 2489.8	19272.8 ± 2752.7^‡^	16404.4 ± 2516.8^*†^	< 0.001
RSMI (kg/m^2^)	8.6 ± 0.9	8.0 ± 1.1	7.4 ± 0.9^*†^	< 0.001
Body fat (%)	34.6 ± 7.4	32.4 ± 8.0	28.3 ± 6.7^*^	0.008

*P < 0.05 versus normal BMD; †P < 0.05 versus osteopenia BMD; ‡P < 0.05 versus osteoporosis BMD; DAS-28 = Disease activity score 28; HAQ = Health Assessment Questionnaire; BMI = Body mass index; RSMI = Relative Skeletal Muscle Index; ANCOVA (analysis of covariance) = adjustments for age, disease duration and cumulative prednisone; SD = standard deviation.

**Table 2.** Dietary intake and biochemical parameters of the study population, according to bone mineral density

Variable	Normal BMD	Osteopenia BMD	Osteoporosis BMD	P
	n = 24	n = 38	n = 21	
	Mean (SD)	Mean (SD)	Mean (SD)	
Energy (kcal)	1421.1 ± 336.3	1528.7 ± 439.5	1646.0 ± 436.4	0.195
Carbohydrate (%)	59.5 ± 5.1	55.2 ± 6.6^*^	52.8 ± 5.5	0.004
Protein (%)	16.2 ± 2.3	17.0 ± 3.3	15.2 ± 2.8	0.527
Fat (%)	24.2 ± 5.5^†^	29.1 ± 5.1^*†^	26.6 ± 5.1^‡^	0.006
w-3 (g/day)	1.0 ± 0.5	1.0 ± 0.4	1.1 ± 0.4	0.463
w-6 (g/day)	6.3 ± 3.3	7.1 ± 2.5	7.2 ± 2.4	0.450
w-6/w-3 (g/day)	6.3 ± 1.4	7.2 ± 1.5^*^	6.7 ± 1.2	0.045
Calcium (mg/day)	597.3 ± 281.7	655.3 ± 254.0	696.2 ± 217.1	0.492
Vitamin D (IU/day)	60.7 ± 56.9	66.6 ± 62.3	88.1 ± 89.6	0.148

*P < 0.05 versus osteoporosis BMD; †P < 0.05 versus osteopenia; ‡P < 0.05 versus normal BMD; ω -3: omega-3; ω -6: omega-6. Protein, fat, carbohydrate, calcium and phosphorus were adjusted for energy. ANCOVA (analysis of covariance): adjustments for age, disease duration and cumulative prednisone. SD = standard deviation.

**Table 3.** Biochemical parameters of the study population, according to bone mineral density

Variable	Normal BMD	Osteopenia BMD	Osteoporosis BMD	P
	n = 24	n = 38	n = 21	
	Mean (SD)	Mean (SD)	Mean (SD)	
Serum total calcium (mg/dl)	9.0 ± 0.6	8.4 ± 0.7^*^	8.7 ± 0.7	0.024
Serum phosphorus (mg/dl)	3.7 ± 0.9	3.4 ± 0.7	3.7 ± 1.2	0.309
Serum intact PTH (pg/ml)	28.9 ±10.3	34.5 ±11.0	32.9 ± 8.9	0.142
Serum osteoprotegerin (pg/ml)	78.6 ± 79.9	72.5 ± 48.8	122.6 ± 87.6^†,‡^	0.036

*P < 0.05 versus osteoporosis BMD; †P < 0.05 versus normal BMD; ‡P < 0.05 versus osteopenia; SD = standard deviation. ANCOVA (analysis of covariance): adjustments for age, disease duration and cumulative prednisone.

**Table 4.** Significant correlations between bone mineral density and different variables among the women with rheumatoid arthritis

Variable	L1-L4 (g/cm^2^) BMD	Femoral neck (g/cm^2^) BMD	Total femur (g/cm^2^) BMD
	r (P)	r (P)	r (P)
Age (years)	-0.37 (0.001)	-0.42 (< 0.001)	-0.35 (0.001)
Disease duration (years)	-0.41 (< 0.001)	-0.23 (0.034)	-0.26 (0.016)
BMI (kg/m^2^)	0.39 (< 0.001)	0.44 (< 0.001)	0.49 (< 0.001)
Total fat-free mass (kg)	0.36 (0.001)	0.53 (< 0.001)	0.57 (< 0.001)
Appendicular lean mass (kg)	0.37 (0.001)	0.54 (< 0.001)	0.59 (< 0.001)
RSMI (kg/m^2^)	0.31 (0.004)	0.47 (< 0.001)	0.54 (< 0.001)
Body fat (%)	0.33 (0.002)	0.30 (0.005)	0.31 (0.004)
Work index (score)	0.32 (0.003)	0.36 (0.001)	0.38 (< 0.001)
Total physical activity index (score)	0.23 (0.033)	0.20 (0.07)	0.26 (0.018)
Fat (%)	-0.29 (0.007)	-0.23 (0.039)	-0.22 (0.047)
iPTH (pg/ml) n = 80	-0.11 (0.326)	-0.24 (0.032)	-0.19 (0.099)

L1-L4 = lumbar spine vertebral 1 to 4; BMD = bone mineral density; BMI = body mass index; RSMI = relative skeletal muscle index; iPTH = intact parathyroid hormone. Pearson’s correlation.

**Table 5.** Regression equations for estimates at the bone sites evaluated among the women with rheumatoid arthritis

Equations	R	R^2^ adjusted	P
Lumbar spine BMD model = 1.153 + 0.005 (W) - 0.005 (D) - 0.004 (A) - 0.003 (FI)	0.626	0.360	0.000
Femoral neck BMD model = 0.764 + 0.000 (ALM) - 0.005 (A) - 0.005 (D) - 0.003 (PTH)	0.698	0.460	0.000
Total femur BMD model = 0.552 + 0.06 (W) + 0.019 (TPA) - 0.05 (A) - 0.003 (D)	0.711	0.481	0.000

BMD = bone mineral density; W = weight; D = disease duration; A = age; FI = fat intake (%); ALM = appendicular lean mass; PTH = parathyroid hormone; TPA = total physical activity.

## DISCUSSION

This was the first study to evaluate the relationship between nutritional factors and body composition in relation to bone mineral density among rheumatoid arthritis patients. Fat intake, age, disease duration and serum iPTH were variables associated with low bone mass among female rheumatoid arthritis patients.

Body composition and age have been associated with BMD. Lean mass and fat mass play a positive role in relation to bone mass. The association between lean mass and bone mass may be due to mechanical load forces on bone. Moreover, fat mass is metabolically active, and increases BMD through hormonal metabolism of adipocytes, thereby intervening in osteoblast/osteoclast function.^11^

In the present study, osteoporotic women had significantly lower fat and lean mass. In part, this may be explained by the inflammatory process, with production of cytokines such as TNFa and higher protein breakdown.^36^^,^^37^ One efficient way to ensure adequate lean mass in the body composition is through physical activity. The low physical activity levels among rheumatoid arthritis patients may contribute towards losses in bone and lean mass. In fact, physical activity levels were related to BMD among our patients, since a positive correlation was observed between the physical activity score and the lumbar spine and total femur BMD, thus suggesting that an improvement in physical activity levels could have a beneficial effect.

The relationship between age and bone loss among rheumatoid arthritis patients has also been observed by another investigator.^38^ This relationship was expected, because older women have been exposed for greater lengths of time to risk factors for bone loss such as corticosteroid use, lower estrogen levels, prolonged immobilization and greater inflammatory state. There is disagreement concerning the effects of glucocorticoids on bone mass in rheumatoid arthritis cases.^38^^,^^39^ In the present study, glucocorticoids had no association with BMD: this may have been due to the low dose administered. Moreover, the osteopenic women showed higher cumulative prednisone levels. In a study by Dykman^40^ using single-photon absorptiometry and previous corticosteroid therapy, multivariate analysis on BMD data clearly demonstrated clearly that the cumulative dose of prednisolone was the most important factor determining corticosteroid osteopenia.

Energy intake did not present significant differences between the groups, and no changes were observed in physical activity levels. Furthermore, patients with reduced bone mass presented low body fat and lean mass, thereby indicating that these patients might have an abnormal basal metabolic rate.

Stone et al.^8^ observed similar fat intake in rheumatoid arthritis patients. Inverse correlations between fat intake and BMD could be explained in terms of increased saturated and trans fat levels, thereby causing increases in oxidative stress. Thus, free radicals may increase osteoclastogenesis and bone resorption through activation of the receptor activator of nuclear factor kappa B (RANK) ligand (RANKL).^14^

No significant associations were observed between calcium, vitamin D and fatty acid intakes and BMD. Calcium and vitamin D intakes were lower than the proposed values for bone loss prevention.^34^ In this regard, natural food sources are scarce and Brazilian foods are not fortified with vitamin D. Studies conducted among Brazilian populations have observed lower levels of circulating 25(OH)D,^41^ thus indicating that sun exposure and diet alone are not enough to maintain vitamin D adequacy. However, prospective follow-up studies are needed in order to determine the calcium and vitamin D intake levels needed to maintain adequate bone metabolism in rheumatoid arthritis patients.

The average w-3 fatty acid intake in all groups was adequate. On the other hand, the opposite was observed for w-6 fatty acids. Despite this, the w-6/w-3 fatty acid ratio found in the present study was inadequate in all groups evaluated. Over recent years, there has been a marked increase in the consumption of w-6 fatty acids and the w-6/w-3 ratio has increased dramatically.^42^ For rheumatoid arthritis patients, the recommended w-6/w-3 ratio is between 2:1 and 3:1, in order to suppress the inflammation.^23^^,^^24^ In our population, a higher ratio was observed, and this might increase the production of inflammatory cytokines, since w-6 fatty acids are involved in the synthesis of prostaglandin E2, while w-3 fatty acids inhibit its production.^17^^,^^43^^,^^44^

Serum osteoprotegerin, iPTH, calcium and phosphorus were within the normal range in all the groups evaluated. The osteopenic women presented lower serum calcium and higher iPTH compared with the other groups. iPTH was negatively and significantly associated with bone mass in the linear regression model for the femoral neck. Other authors^45^^,^^46^ have found negative correlations between PTH and BMD among rheumatoid arthritis patients. PTH therapy directly stimulates osteoblastogenesis and inhibits osteoblast apoptosis. Recently, Saag et al.^47^ demonstrated that teriparatide had significant skeletal benefits (increased BMD and decreased new vertebral fractures) among patients with glucocorticoid-induced osteoporosis.

Osteoprotegerin levels are frequently higher in individuals with osteoporosis,^48^ as was observed among our patients. Masi et al.^48^ suggested that patients with greater disease severity could have higher levels of serum osteoprotegerin because of a compensatory self-defense response for keeping control over the immune mechanisms that are responsible for bone and cartilage destruction. Recent data have indicated that denosumab, a fully human monoclonal antibody against RANKL, is able to inhibit RANKL-RANK interaction, thereby mimicking the endogenous effects of osteoprotegerin. Thus, it may be used to treat osteoporosis and periarticular bone loss and to decrease bone erosions in rheumatoid arthritis cases, although this antagonism does not treat synovitis.^49^

The present study has some limitations, such as the absence of measurements on basal energy expenditure, biochemical markers for pro-inflammatory cytokines and serum 25(OH)D, given that the changes in bone mass and body composition were due to inflammatory processes. In addition, osteoporotic fractures were not evaluated. Although the design of this study only allowed hypotheses of associations to be raised, it was demonstrated that body composition and nutrient intake were associated with BMD among patients with rheumatoid arthritis. Thus, health professionals should include nutritional and physical activity advice in the management of rheumatoid arthritis patients.

## CONCLUSION

In summary, the present study indicates that nutritional factors and body composition are important modifiable factors that can improve bone mass and prevent osteoporotic fractures in rheumatoid arthritis patients.
